# Prenylated Diphenyl Ethers from the Marine Algal-Derived Endophytic Fungus *Aspergillus tennesseensis*

**DOI:** 10.3390/molecules23092368

**Published:** 2018-09-17

**Authors:** Zhao-Xia Li, Xiu-Fang Wang, Guang-Wei Ren, Xiao-Long Yuan, Ning Deng, Gui-Xia Ji, Wei Li, Peng Zhang

**Affiliations:** 1 College of Marine Life Sciences, Ocean University of China, Qingdao, Shandong 266003, China; zhaoxiali0503@163.com; 2 Tobacco Research Institute, Chinese Academy of Agricultural Sciences, Qingdao, Shandong 266101, China; wangxiufang02@caas.cn (X.-F.W.); renguangwei@caas.cn (G.-W.R.); rayrock@126.com (X.-L.Y.); dnsmile0208@163.com (N.D.); 18853815579@163.com (G.-X.J.)

**Keywords:** marine-derived fungi, *Aspergillus tennesseensis*, secondary metabolites, diphenyl ethers, antimicrobial activity, cytotoxicity

## Abstract

Considerable attention has been paid to marine derived endophytic fungi, owing to their capacity to produce novel secondary metabolites with potent bioactivities. In this study, two new compounds with a prenylated diphenyl ether structure—diorcinol L (**1**) and (*R*)-diorcinol B (**2**)—were isolated from the marine algal-derived endophytic fungus *Aspergillus tennesseensis*, along with seven known compounds: (*S*)-diorcinol B (**3**), 9-acetyldiorcinol B (**4**), diorcinol C (**5**), diorcinol D (**6**), diorcinol E (**7**), diorcinol J (**8**), and a dihydrobenzofuran derivative **9**. Their structures were elucidated by extensive NMR spectroscopy studies. Compound **2** represents the first example of an *R*-configuration in the prenylated moiety. All these isolated compounds were examined for antimicrobial and cytotoxic activities. Compounds **1**–**9** exhibited antimicrobial activities against some human- and plant-pathogenic microbes with MIC values ranging from 2 to 64 μg/mL. Moreover, compound **9** displayed considerable inhibitory activity against the THP-1 cell line in vitro, with an IC_50_ value of 7.0 μg/mL.

## 1. Introduction

Numerous structurally diverse diphenyl ether derivatives have been previously isolated and characterized from different microorganisms, especially from filamentous fungi like *Aspergillus* sp. [1,2,3], *Penicillium* sp. [4], and *Acremonium* sp. [5]. Compounds of this family are reported to possess a wide range of interesting biological activities, such as antibacterial [4], cytotoxic [6], DPPH radical-scavenging [7], enzyme inhibitory [8], and antiviral [9] activities.

**Figure 1:**
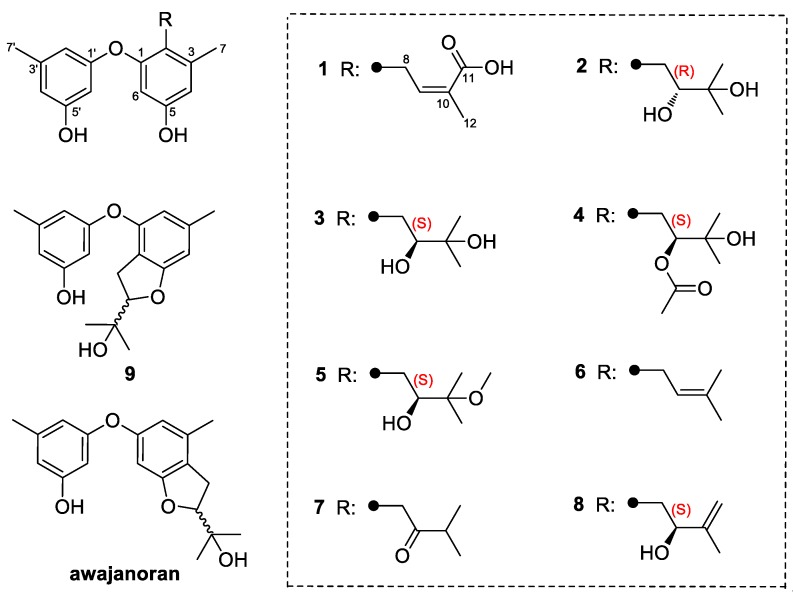
Structures of the isolated compounds **1**–**9** and the related compound awajanoran.

Marine-derived microbes, particularly endophytic fungi, are well known for their ability to synthesize secondary metabolites with novel skeletons and diverse biological activities [10]. Marine endophytic fungi are commonly distributed in many marine habitats, specifically in marine plants including algae and mangrove [11]. These microorganisms spend the whole or part of their lifetime residing inter-and/or intra-cellularly living tissues of the host without causing any apparent symptoms of disease. Although their exact role is not well understood, endophytes have been proved to possess a high potential to produce novel bioactive secondary metabolites. To date, a large number of compounds have been isolated and identified, with a wide range of biological activities including cytotoxic, antimicrobial, antiviral, and antioxidative activities [12]. In this study, a fungal strain *Aspergillus tennesseensis* was isolated from the fresh inner tissue of an unidentified marine algae. After a surface sterilization procedure, the fungal strain was isolated and described as an endophytic fungus [13]. The ethyl acetate (EtOAc) extracts of its culture broth were analyzed by HPLC, and a series of peaks with similar UV absorptions at approximately 205 and 280 nm were detected. We thus established a large-scale culture of this fungus in liquid medium. Subsequent chemical investigation led to the isolation of two new prenylated diphenyl ethers—diorcinol L (**1**) and (*R*)-diorcinol B (**2**)—along with seven known analogues: (*S*)-diorcinol B (**3**), 9-acetyldiorcinol B (**4**), diorcinol C (**5**), diorcinol D (**6**), diorcinol E (**7**), diorcinol J (**8**), and a dihydrobenzofuran derivative **9** (Figure 1). Compounds **1**–**8** belong to the diorcinol family, and it appears that the new compound **2** is the first example of an *R*-configuration in the diorcinol family. Herein, we report the isolation, structure elucidation, and bioactivities of these isolated compounds.

## 2. Results and Discussion

### 2.1. Structural Elucidation of the New Compounds

**Figure 2:**
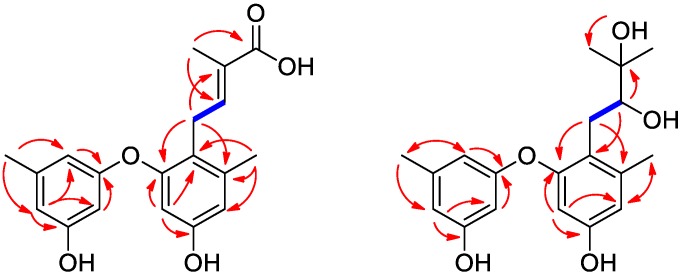
Key COSY (bold lines) and HMBC (arrows) correlations for compounds **1** and **2**.

Compound **1** was obtained as a light yellow amorphous powder, and its molecular formula C_19_H_20_O_5_ was established from a prominent pseudomolecular ion peak at *m*/*z* 329.1377 [M + H]^+^ in its HRESIMS (Figure S1). After examining the ^1^H data (Table 1) and the HSQC spectrum (Figure S5), signals were attributed to one prenyl group at δ_H_ 3.17 (2H, d, *J* = 7.0 Hz) and 6.17 (1H, t, *J* = 7.0 Hz), three singlet methyl groups at δ_H_ 1.73 (3H, s, H-12), 2.13 (3H, s, H-7), and 2.14 (3H, s, H-7′), and five aromatic methines at δ_H_ 6.08 (1H, br s, H-6′), 6.10 (1H, d, *J* = 2.1 Hz, H-6), 6.11 (1H, br s, H-4′), 6.26 (1H, br s, H-2′), and 6.39 (1H, d, *J* = 2.1 Hz, H-4). The pattern of the aromatic signals indicated the presence of a 1,3,5-trisubstituted benzene and a 1,2,3,5-tetrasubstituted benzene system. The ^13^C NMR spectrum (Table 1) combined with the DEPT experiments (Figure S3) displayed 19 signals consisting of three methyls, one methylene, six aromatic/olefinic methines (with four oxygen-bearing ones), one carboxyl, and eight aromatic/olefinic quaternary carbons. These data suggested that compound **1** contains a diphenyl ether unit, which was also supported by the HMBC correlations (Figure 2 and Figure S6). Detailed analysis of the NMR data of **1** revealed some structural similarities to diorcinol D (**6**), which was previously isolated from a marine-derived fungus *Aspergillus versicolor* ZLN-60 [2]. However, the signals of a methyl resonating at δ_H_/δ_C_ 1.59 (H-11)/26.0 (C-11) in diorcinol D were absent in the NMR spectra of **1**. Instead, a carboxyl group was observed. The above information indicated that compound **1** was the carboxylic acid derivative of diorcinol D, and a trivial name, diorcinol L, was assigned to this compound.

Compound **2** was also obtained as yellowish powder. Its molecular formula was determined to be C_19_H_24_O_5_ by HRESIMS (*m*/*z* 333.1689 [M + H]^+^, calcd for C_19_H_25_O_5_, 333.1697) (Figure S7). Comparison of the NMR data of **2** (Figures S8–S12) with those of diphenyl ethers indicated they shared a common structural core. Thus, the structural elucidation of **2** was quite straightforward due to the close relationships with the other related compounds. Compound **2** and (*S*)-diorcinol B (**3**) [2] displayed almost superimposed resonances except for the C-9 in the upfield region. The chemical shift of C-9 in compound **2** was 73.2 ppm, whereas in diorcinol B, the chemical shift of C-9 was 78.7 ppm [2]. This significant difference implied that compounds **2** and **3** were diastereomers caused by the atropisomerism around the diphenyl ring. Since **3** had 9*S*-configuration, compound **2** was tentatively proposed to have 9*R*-configuration, which was supported by the opposite specific rotation data (+23.2 in **2** vs. −45.1 in **3**). As for the absolute configuration, so far all of the reported diorcinols showed negative optical rotation values indicating the 9*S*-configuration, and this is thus the first report of a positive rotation indicating the *R*-configuration of C-9.

Besides the new compounds, (*S*)-diorcinol B (**3**) [2], 9-acetyldiorcinol B (**4**) [14], diorcinol C (**5**) [2], diorcinol D (**6**) [2], diorcinol E (**7**) [2], diorcinol J (**8**) [15], and a dihydrobenzofuran derivative, 3-(2-(1-hydroxy-1-methyl-ethyl)-6-methyl-2,3-dihydrobenzofuran-4-yloxy)-5-methylphenol (**9**) [16] were also isolated from this fungus. Their structures were elucidated by spectroscopy and by comparison with previously reported data. All these compounds are prenylated derivatives of diorcinol [17]. Aside from compounds **1**–**9**, another analogue with the 2,3-dihydrobenzofuran moiety, awajanoran, was isolated from an agar-culture of *Acremonium* sp. [5]. It appeared that the prenylation could arbitrarily occur at positions of C-2, C-4, and C-6. Moreover, awajanoran exhibited moderate cytotoxic activity in contrast to **9**. This indicated that the location of the prenylation could affect the bioactivities to a certain extent.

### 2.2. Biological Activities of the Isolated Compounds

All of the isolated compounds **1**–**9** were evaluated for antimicrobial activity against several human- and plant-pathogenic microbes, as well as for cytotoxicity. Compounds **2** and **8** showed antibacterial activities against *Escherichia coli* and *Bacillus subtilis*, respectively, with a MIC value of 4 μg/mL (Table 2). In particular, compound **9** exhibited promising inhibitory activity against *Bacillus subtilis* with a MIC value of 2 μg/mL. In the antifungal assay, compounds **2** and **7** showed considerable inhibitory effect against *Cochliobolus heterostrophus* and *Gaeumannomyces graminis* with MIC values of 4 and 2 μg/mL, respectively, which were higher than that of the positive control prochloraz (MIC = 8 μg/mL). Notably, **2** was generally more active than 3 in the antimicrobial assay. It could be possibly be explained by the configuration, which should affect the antimicrobial activity, and the *R*-configuration, which could enhance the activity in the diorcinol family.

Compounds **1**–**9** were also evaluated for cytotoxicity against eight tumor cell lines (Du145, HeLa, HepG2, MCF-7, NCI-H460, SGC-7901, SW1990, and U251) in vitro. Compound **9** selectively exhibited cytotoxicity against the THP-1 cell line with the IC_50_ value of 7.0 μg/mL, whereas others displayed weak or no inhibitory activity (IC_50_ > 50 μg/mL, data not shown).

## 3. Materials and Methods

### 3.1. General Experimental Procedures

Optical rotations were measured on a P-1020 digital polarimeter (Jasco, Tokyo, Japan) and UV spectra were recorded with MeOH on a UV-2700 spectrophotometer (Shimadzu, Kyoto, Japan). The ^1^H, ^13^C, and 2D NMR spectra (500 MHz for ^1^H and 125 MHz for ^13^C) were acquired using an DD2 500 MHz NMR spectrometer (Agilent, Santa Clara, CA, USA) with TMS as internal standard. HRESIMS spectra were obtained from a LTQ Orbitrap XL spectrometer (Thermo Scientific, Waltham, MA, USA). Analytical HPLC was performed using a UPLC-class system (Waters, Milford, MA, USA) using a C_18_ column (1.6 μm, 2.1 mm × 50 mm) equipped with a TUV-detector. Column chromatography (CC) was performed with silica gel (100–200 and 200–300 mesh, Qingdao Haiyang Chemical Factory, Qingdao, China), Lobar LiChroprep RP-18 (40–60 μm, Merck, Darmstadt, Germany), and Sephadex LH-20 (Merck). All the solvents purchased were of analytical grade or HPLC grade.

### 3.2. Fungal Material

The fungus *Aspergillus tennesseensis* strain OUCMB I 140430 was isolated by one of the authors (Z.-X.L.) from the fresh inner tissue of an unidentified marine alga, which was collected at Qingdao, China, in July 2014. The fungal strain was described as an endophyte due to the rigorous surface sterilization procedure [13]. The isolate was identified as *A. tennesseensis* based on its morphological characteristics and partial 18S rDNA gene sequence data, with the GenBank (NCBI) accession number MH785494. This strain has been preserved at Tobacco Research Institute, Chinese Academy of Agricultural Sciences.

### 3.3. Fermentation, Extraction and Isolation

For chemical investigations, the fungus was statically cultivated in 100 × 1 L Erlenmeyer flasks for 30 days at 28 °C, each containing 300 mL of the liquid medium (the composition of the medium includes 20 g mannitol, 10 g glucose, 3 g peptone, 5 g yeast extract, and 1000 mL of sterile seawater). The filtrate of the fermented broth (30 L) was extracted repeatedly with EtOAc, while the mycelia (20.6 g) were extracted three times with a mixture of acetone and H_2_O (80%:20%). Thereafter, they were combined to afford a residue (32.8 g), which was subjected to silica gel chromatography using a VLC column with a stepwise gradient of a mixture of petroleum ether (PE): ethyl acetate (EtOAc) (from 5:1 to 1:1) and dichloromethane (DCM): methanol (MeOH) to provide 10 fractions (Fr.1–Fr.10). Fr.3 (2.6 g), eluted with PE:EtOAc (2:1, *v*/*v*), was purified by Sephadex LH-20 (MeOH) to obtain three subfractions (Fr.3.1–Fr.3.3). Fr.3.1 (1.1 g) was further separated by column chromatography (CC) (silica gel, DCM:MeOH gradient, from 30:1 to 20:1) to obtain compounds **1** (8.7 mg) and **2** (5.6 mg); Fr.3.2 (0.2 g) was subjected to preparative thin layer chromatography (pTLC, PE:EtOAc = 1:1) to afford compound **7** (25.3 mg); Fr.3.3 (0.8 g) was purified by semipreparative HPLC (MeOH:H_2_O, 70%) to afford **5** (30.6 mg) and **9** (52.1 mg). Fr.4 (1.6 g), eluted with PE:EtOAc (1:1, *v*/*v*), was separated by Lobar LiChroprep RP-18 from MeOH:H_2_O 3:7 to 8:2, and finally Sephadex LH-20 (MeOH) to afford compounds **3** (12.9 mg), **4** (20.3 mg), **6** (17.2 mg), and **8** (6.6 mg).

*Diorcinol L* (**1**): light yellow amorphous powder; UV (MeOH) *λ*_max_ (log ε) 205 (4.09), 281 (2.91) nm; ^1^H-and ^13^C-NMR data, see Table 1; ESIMS at *m*/*z* 329.13 [M + H]^+^ and 351.12 [M + Na]^+^; HRESIMS at *m*/*z* 329.1377 [M + H]^+^ (calcd for C_19_H_21_O_5_, 329.1384) and 351.1199 [M + Na]^+^ (calcd for C_19_H_20_O_5_Na, 351.1203).

*(R)-Diorcinol B* (**2**): yellowish powder; ${\left[\alpha \right]}_{D}^{25}$+23.2 (*c* 0.1, MeOH); UV (MeOH) *λ*_max_ (log ε) 205 (4.44), 281 (3.30) nm; ^1^H and ^13^C-NMR data, see Table 1; ESIMS at *m*/*z* 333.16 [M + H]^+^; HRESIMS *m*/*z* 333.1689 [M + H]^+^ (calcd for C_19_H_25_O_5_, 333.1697).

### 3.4. Antimicrobial Assay

Antimicrobial assays against the human- and plant-pathogenic bacteria *Bacillus subtilis*, *Escherichia coli*, *Pseudomonas aeruginosa*, *Ralstonia solanacearum* and plant pathogenic fungi *Alternaria alternata*, *Cochliobolus heterostrophus*, *Gaeumannomyces graminis*, *Glomerella cingulata*, *Mucor hiemalis*, and *Thielaviopsis basicola* were carried out using the well diffusion method [18]. Chloramphenicol was used as a positive control for the bacteria, while prochloraz (a common broad-spectrum fungicide usually used in agriculture) was used as a positive control for the fungi.

### 3.5. Cytotoxicity Assay

The cytotoxic activity of the isolated compounds against six tumor cell lines including A549 (human lung adenocarcinoma epithelial cell line), Du145 (human prostate cancer cell line), HeLa (human cervix carcinoma cell line), MCF-7 (human breast adenocarcinoma cell line), MDA-MB-231 (human breast cancer cell line), and THP-1 (human monocytic cell line) were determined according to the previously reported the Cell Counting Kit-8 (CCK-8) colorimetric method [19].

## 4. Conclusions

In conclusion, nine prenylated diphenyl ethers, including two new ones, were isolated and identified from the marine algal-derived endophytic fungus *Aspergillus tennesseensis*. Among them, compound **2** represents the first example of an *R*-configuration in the prenylated moiety. The antimicrobial and cytotoxic activities of the isolated compounds were evaluated, and some of the compounds showed promising activities. These results indicated that the algal-derived endophytic fungi are a prolific resource of novel bioactive natural products.

## Figures and Tables

**Table 1:** ^1^H (500 MHz) and ^13^C-NMR (125 MHz) data of compounds **1** and **2** in DMSO-*d*_6_.

	Compound 1		Compound 2	
No.	δ_H_ (mult, *J* in Hz)	δ_C_, Type	δ_H_ (mult, *J* in Hz)	δ_C_, Type
1		154.8, C		156.1, C
2		121.0, C		119.3, C
3		139.1, C		139.8, C
4	6.39, d (2.1)	113.5, CH	6.37, d (2.2)	113.1, CH
5		156.5, C		156.6, C
6	6.10, d (2.1)	104.8, CH	6.08, d (2.2)	104.1, CH
7	2.13, s	19.8, CH_3_	2.25, s	20.5, CH_3_
8	3.17, d (7.0)	25.6, CH_2_	3.05, dd (14.5, 2.0) 2.80, dd (14.5, 11.4)	29.3, CH_2_
9	6.17, t (7.0)	132.5, CH	4.04, dd (11.4, 2.0)	73.2, CH
10		133.8, C		72.1, C
11		172.5, C	1.19, s	27.2, CH_3_
12	1.73, s	14.1, CH_3_	1.20, s	26.2, CH_3_
1’		159.1, C		158.9, C
2’	6.26, br s	111.0, CH	6.30, br s	111.2, CH
3’		140.2, C		140.4, C
4’	6.11, br s	109.0, CH	6.20, br s	109.9, CH
5’		158.9, C		158.4, C
6’	6.08, br s	102.3, CH	6.10, br s	102.7, CH
7’	2.14, s	21.6, CH_3_	2.16, s	21.6, CH_3_
10-OH			4.77, s	

**Table 2:** Minimum inhibitory concentration (MIC, μg/mL) of compounds **1**–**9** against human- and plant-pathogenic microbes.

Compound	Bacteria ^a^				Fungi ^b^					
	B. s.	E. c.	P. a.	R. s.	A. a.	C. h.	G. g.	G. c.	M. h.	T. b.
1	− ^c^	8	16	8	16	32	16	32	−	−
2	8	4	16	16	64	4	16	64	32	64
3	−	−	16	32	−	−	32	64	64	64
4	16	16	32	8	−	−	32	−	32	64
5	8	−	32	−	−	−	−	32	16	−
6	32	8	64	−	−	−	64	32	−	−
7	8	64	−	32	−	−	2	−	16	−
8	4	−	64	16	−	−	−	−	8	−
9	2	64	32	−	−	−	−	16	8	8
Ch ^d^	0.5	2	16	16						
Pr ^e^					16	8	8	32	8	64

^a^ B. s., *Bacillus subtilis*; E. c., *Escherichia coli*; P. a., *Pseudomonas aeruginosa*; R. s., *Ralstonia solanacearum*; ^b^ A. a., *Alternaria alternate*; C. h., *Cochliobolus heterostrophus*; G. g., *Gaeumannomyces graminis*; G. c., *Glomerella cingulata*; M. h., *Mucor hiemalis*; T. b., *Thielaviopsis basicola*; ^c^ MIC > 64 μg/mL; ^d^ Ch, positive control, chloramphenicol; ^e^ Pr, positive control, prochloraz.

## Supplementary Materials

The following are available online. Figures S1–S12: HRESIMS and NMR spectra of compounds **1** and **2**.
