# Supplementary material for: Prenylated Diphenyl Ethers from the Marine Algal-Derived Endophytic Fungus Aspergillus tennesseensis
Source: Molecules. 2018 Sep 17;23(9):2368. doi: 10.3390/molecules23092368 (PMC6225247; doi:10.3390/molecules23092368)
Supplement: Supplementary file 1 [file molecules-23-02368-s001.pdf]

Supplementary Material

Prenylated Diphenyl Ethers from the Marine Algal-Derived Endophytic Fungus *Aspergillus tennesseensis*

Zhao-Xia Li [1,†], Xiu-Fang Wang [2,†], Guang-Wei Ren [2], Xiao-Long Yuan [2], Ning Deng [2], Gui-Xia Ji [2],Wei Li [1,*], and Peng Zhang [2,*]

[1] College of Marine Life Sciences, Ocean University of China, Qingdao, Shandong 266003, China; 770470311@qq.com (Z.-X. L.)

[2] Tobacco Research Institute, Chinese Academy of Agricultural Sciences, Qingdao, Shandong 266101, China; wangxiufang02@caas.cn (X.-F. W.); renguangwei@caas.cn (G.-W. R.); rayrock@126.com (X.-L. Y.); 297207121@qq.com (N. D.); 871721831@qq.com (G.-X. J.)

† These authors contributed equally to this work

* Correspondence: liwei01@ouc.edu.cn (W. L.); zhangpeng@caas.cn (P. Z.); Tel: +86-532-66715079

Table of Contents

Figure S1. HRESIMS spectrum of compound **1**;

Figure S2. ^{1}H NMR (500 MHz, DMSO-d_6) spectrum of compound **1**;

Figure S3. ^{13}C NMR (125 MHz, DMSO-d_6) and DEPT spectra of compound **1**;

Figure S4. COSY spectrum of compound **1**;

Figure S5. HSQC spectrum of compound **1**;

Figure S6. HMBC spectrum of compound **1**;

Figure S7. HRESIMS spectrum of compound **2**;

Figure S8. ^{1}H NMR (500 MHz, DMSO-d_6) spectrum of compound **2**;

Figure S9. ^{13}C NMR (125 MHz, DMSO-d_6) and DEPT spectra of compound **2**;

Figure S10. COSY spectrum of compound **2**;

Figure S11. HSQC spectrum of compound **2**;

Figure S12. HMBC spectrum of compound **2**.

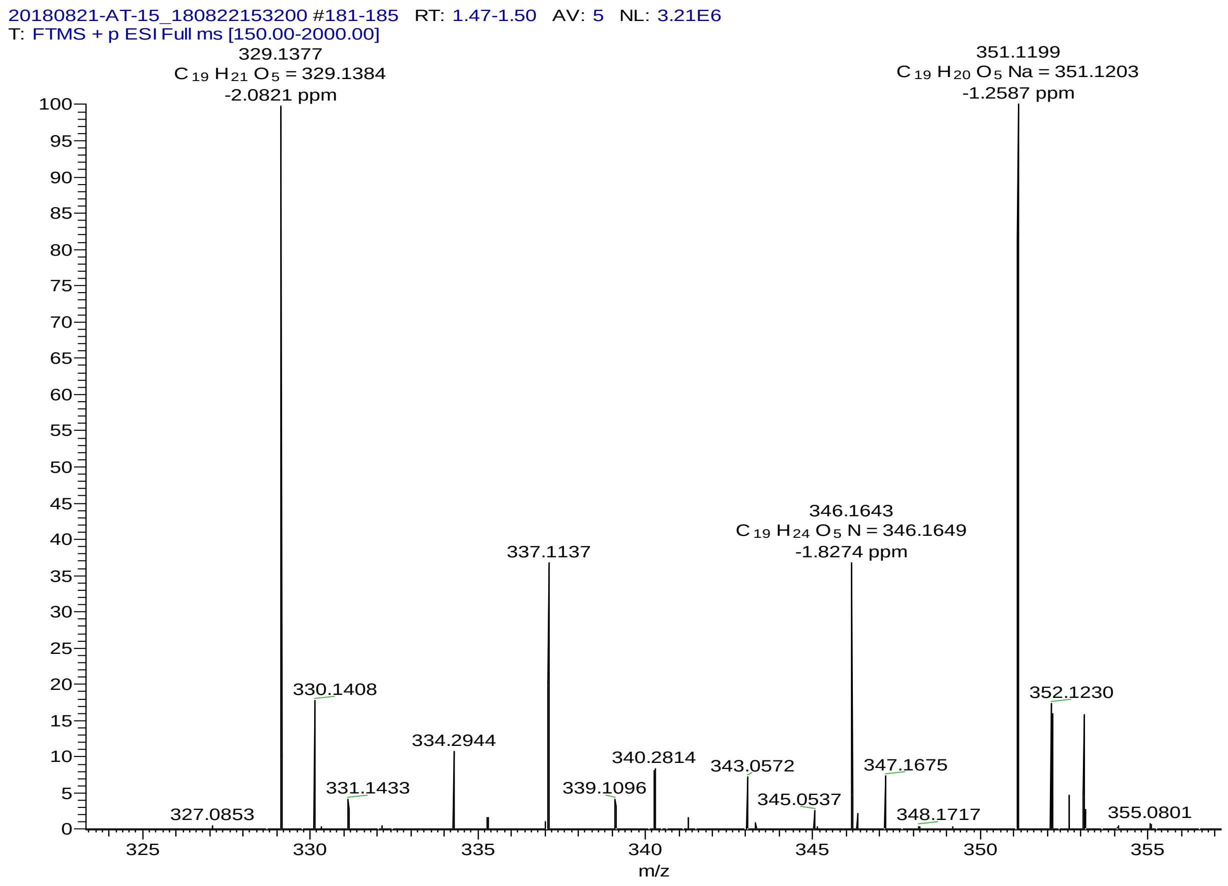

Figure S1. HRESIMS spectrum of compound **1**

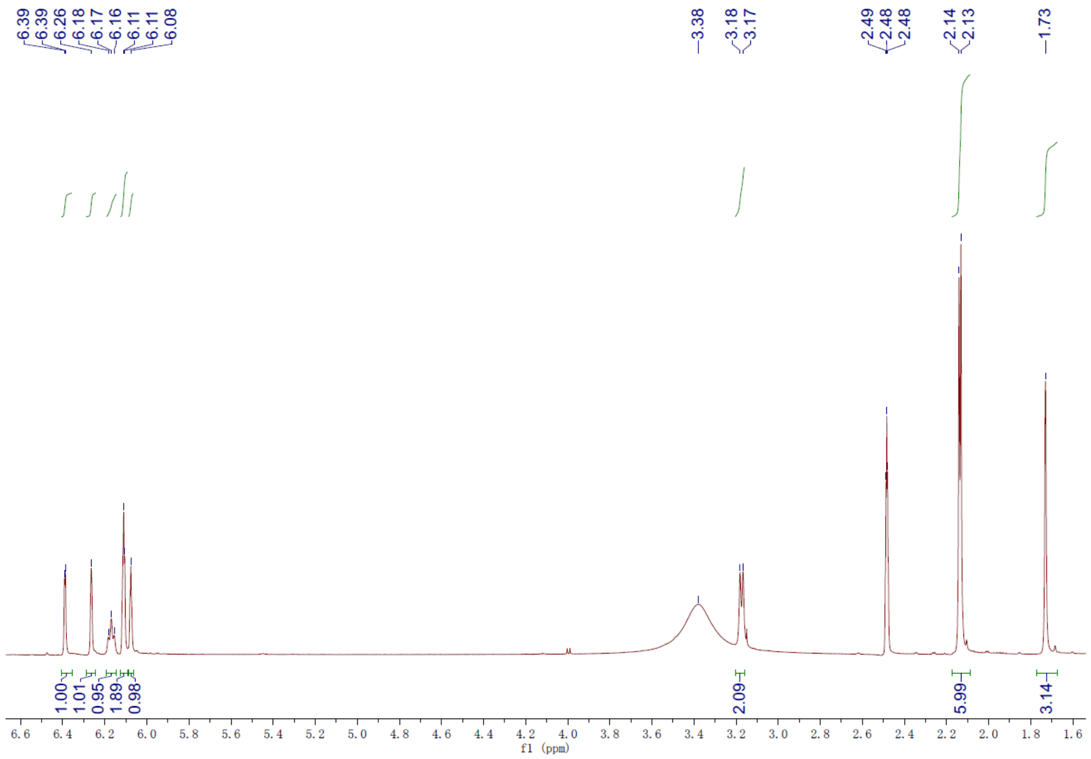

Figure S2. ^{1}H NMR (500 MHz, DMSO-d_6) spectrum of compound **1**

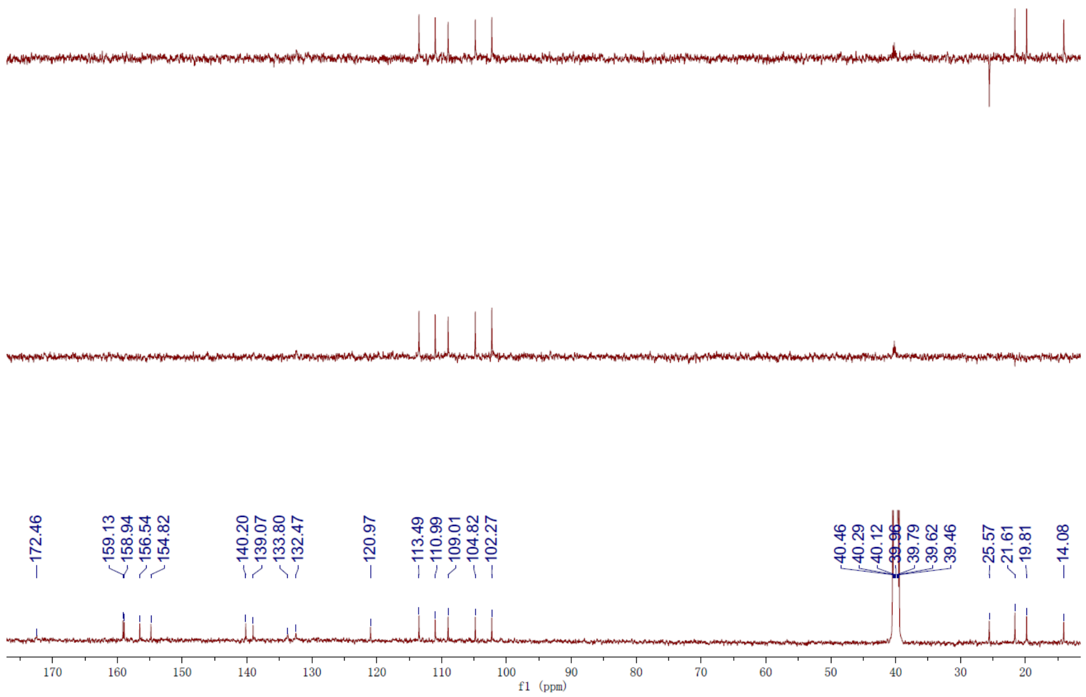

Figure S3. ^{13}C NMR (125 MHz, DMSO-d_6) and DEPT spectra of compound **1**

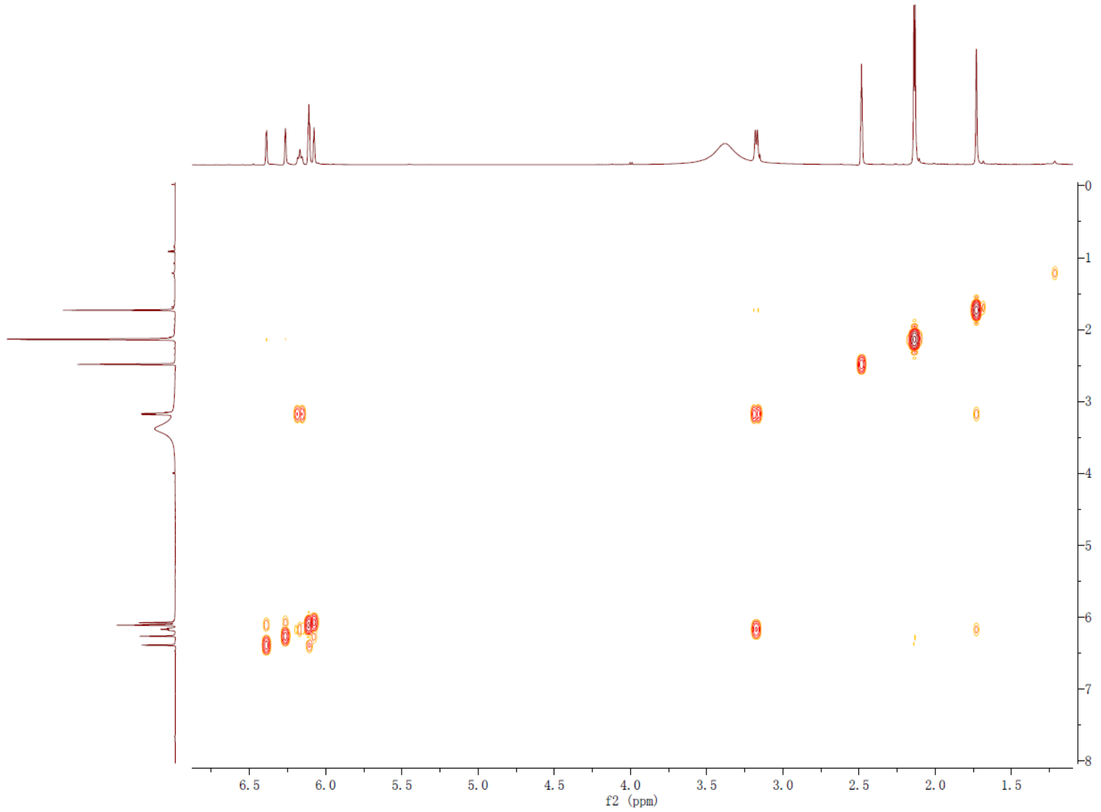

Figure S4. COSY spectrum of compound **1**

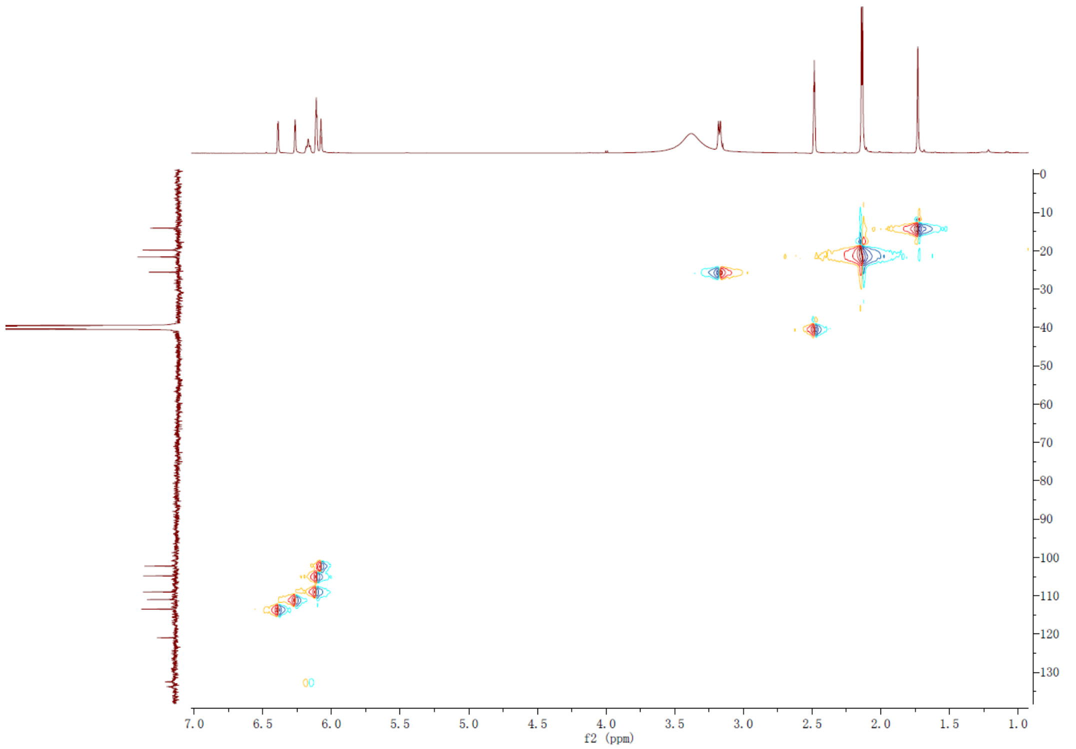

Figure S5. HSQC spectrum of compound **1**

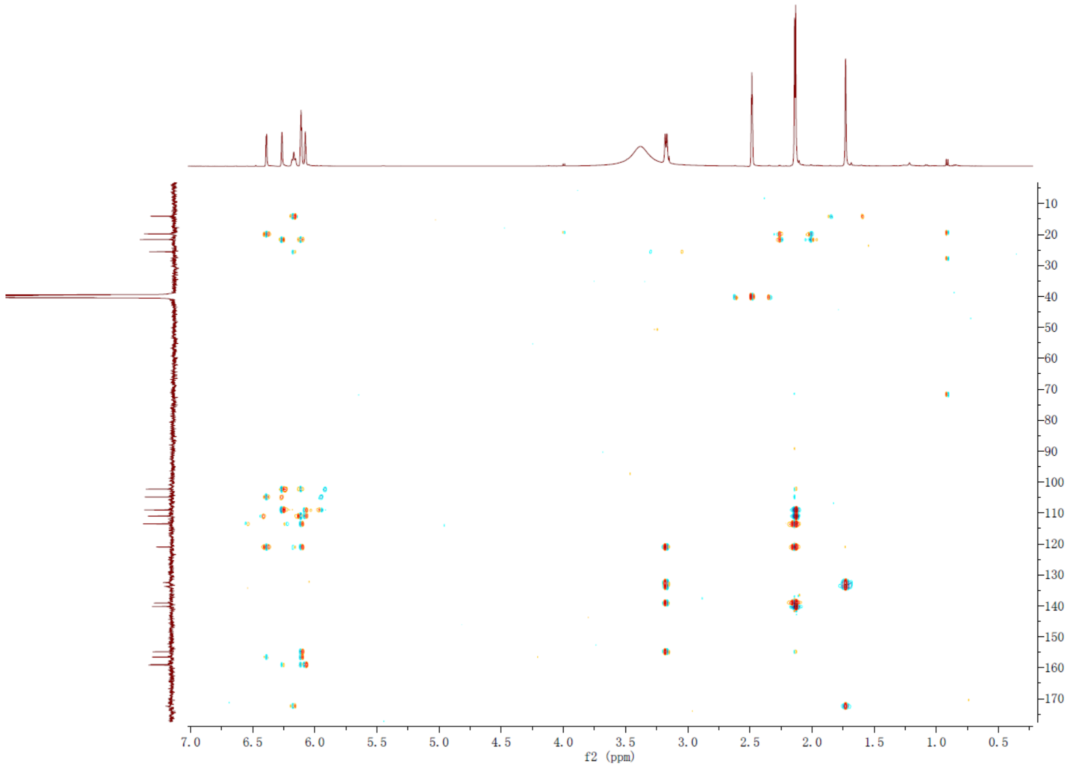

Figure S6. HMBC spectrum of compound **1**

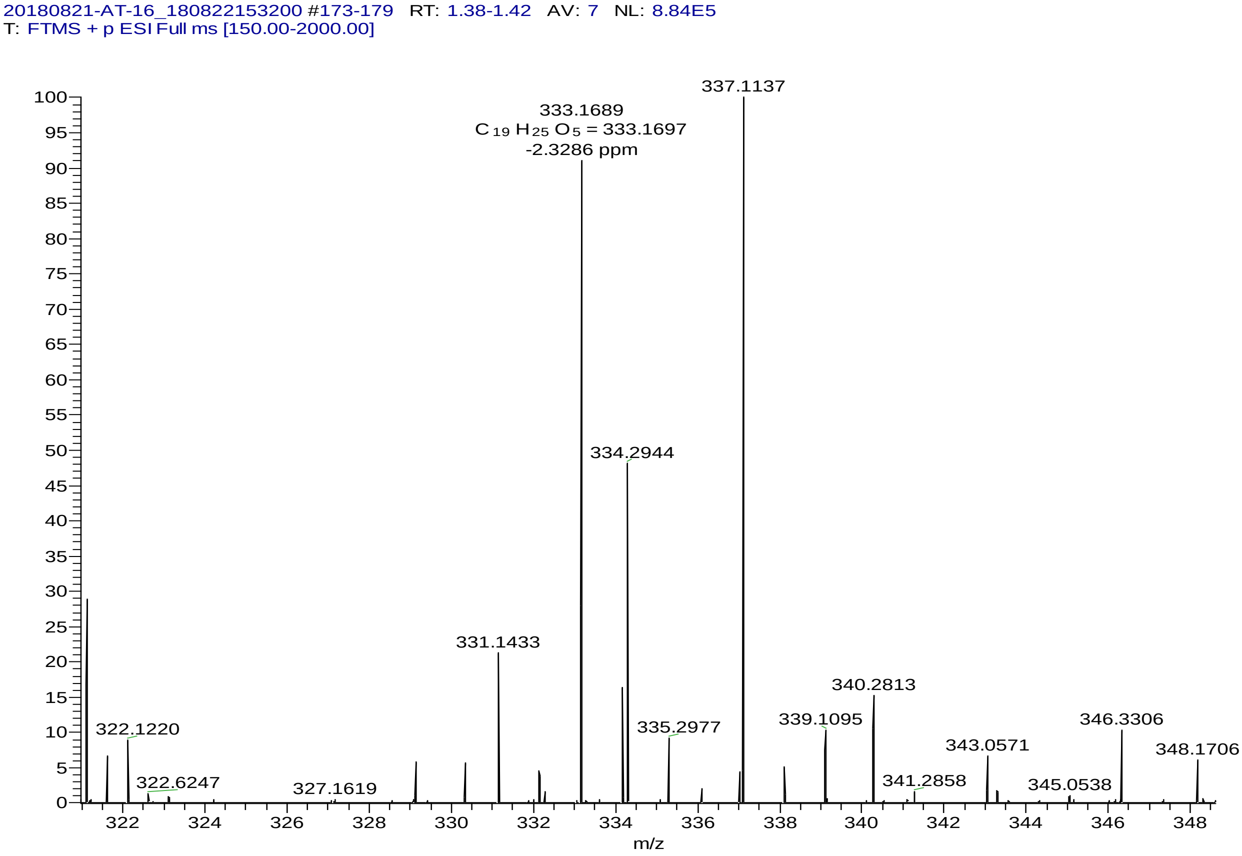

Figure S7. HRESIMS spectrum of compound **2**

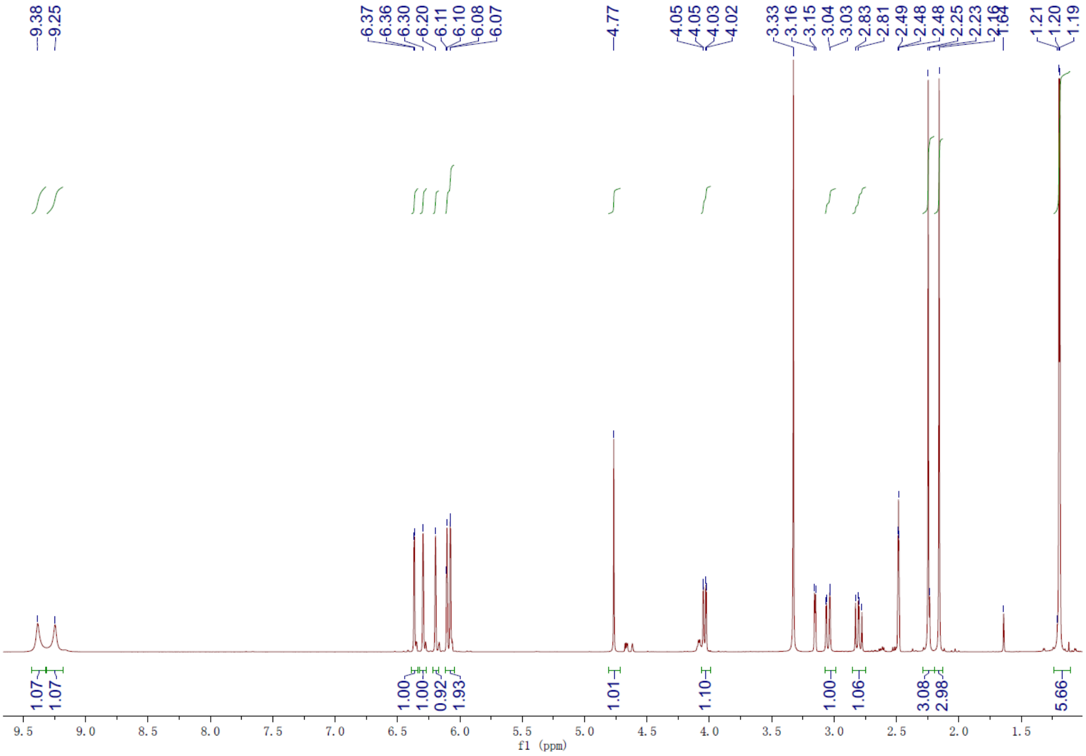

Figure S8. ^{1}H NMR (500 MHz, DMSO-d_6) spectrum of compound **2**

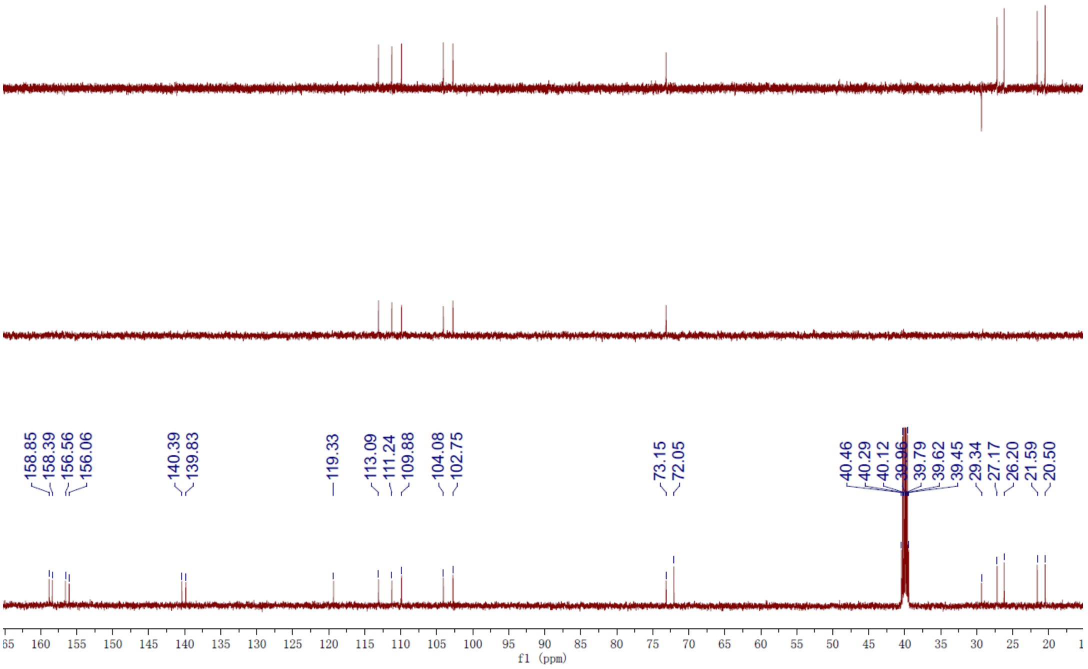

Figure S9. ^{13}C NMR (125 MHz, DMSO-*d*6) and DEPT spectra of compound **2**

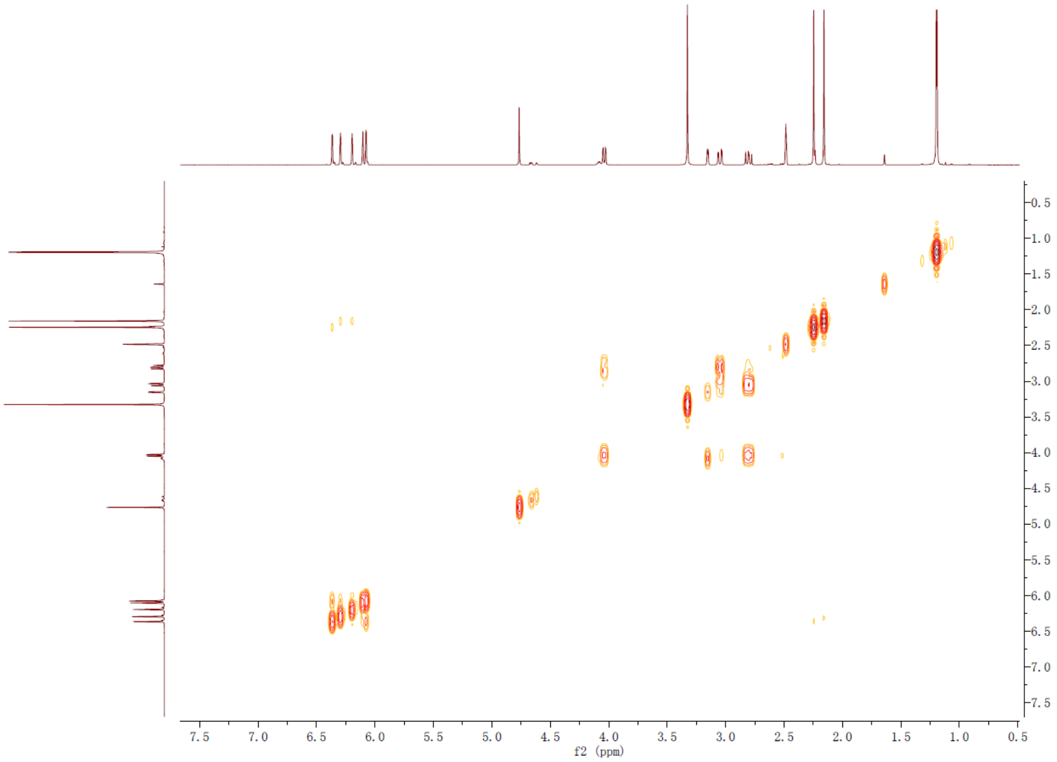

Figure S10. COSY spectrum of compound **2**

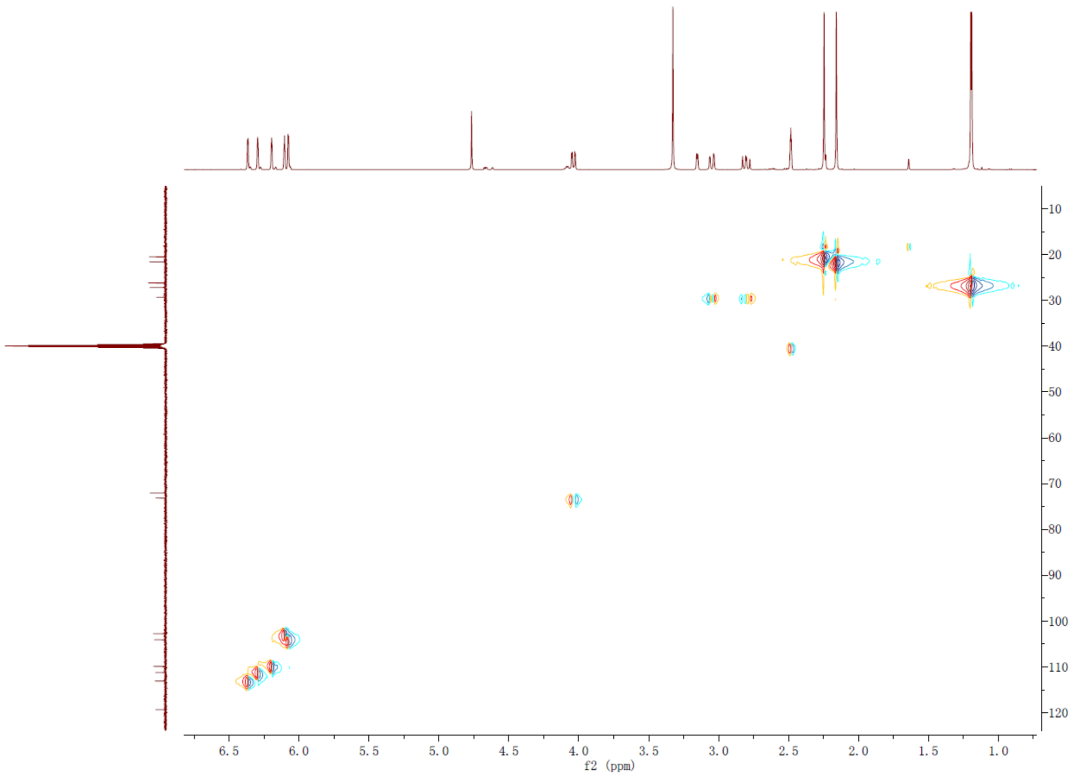

Figure S11. HSQC spectrum of compound **2**

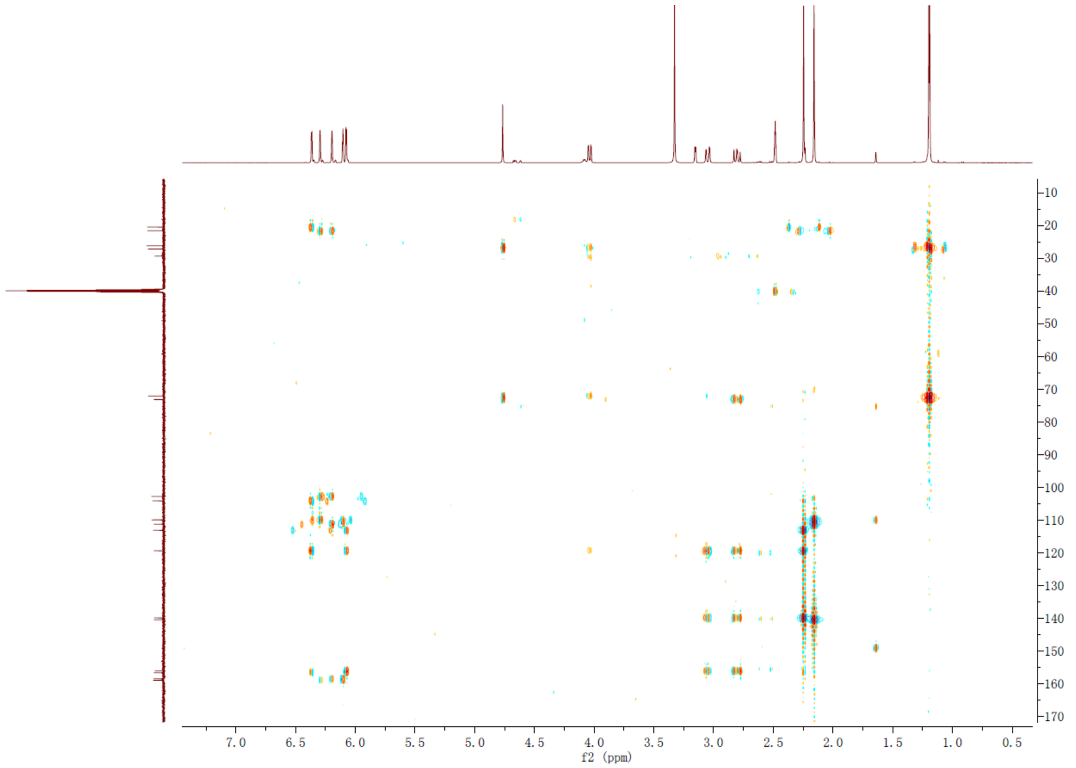

Figure S12. HMBC spectrum of compound **2**
